# Molecular subtype identification and prognosis stratification based on golgi apparatus-related genes in head and neck squamous cell carcinoma

**DOI:** 10.1186/s12920-024-01823-9

**Published:** 2024-02-16

**Authors:** Aichun Zhang, Xiao He, Chen Zhang, Xuxia Tang

**Affiliations:** Department of Otolaryngology, The First Affiliated Hospital of Zhejiang Chinese Medical University (Zhejiang Provincial Hospital of Traditional Chinese Medicine), 310006 Hangzhou, Zhejiang Province P. R. China

**Keywords:** Golgi apparatus, Head and neck squamous cell carcinoma, Consensus clustering, Prognosis, Nomogram

## Abstract

**Background:**

Abnormal dynamics of the Golgi apparatus reshape the tumor microenvironment and immune landscape, playing a crucial role in the prognosis and treatment response of cancer. This study aims to investigate the potential role of Golgi apparatus-related genes (GARGs) in the heterogeneity and prognosis of head and neck squamous cell carcinoma (HNSCC).

**Methods:**

Transcriptional data and corresponding clinical information of HNSCC were obtained from public databases for differential expression analysis, consensus clustering, survival analysis, immune infiltration analysis, immune therapy response assessment, gene set enrichment analysis, and drug sensitivity analysis. Multiple machine learning algorithms were employed to construct a prognostic model based on GARGs. A nomogram was used to integrate and visualize the multi-gene model with clinical pathological features.

**Results:**

A total of 321 GARGs that were differentially expressed were identified, out of which 69 were associated with the prognosis of HNSCC. Based on these prognostic genes, two molecular subtypes of HNSCC were identified, which showed significant differences in prognosis. Additionally, a risk signature consisting of 28 GARGs was constructed and demonstrated good performance for assessing the prognosis of HNSCC. This signature divided HNSCC into the high-risk and low-risk groups with significant differences in multiple clinicopathological characteristics, including survival outcome, grade, T stage, chemotherapy. Immune response-related pathways were significantly activated in the high-risk group with better prognosis. There were significant differences in chemotherapy drug sensitivity and immune therapy response between the high-risk and low-risk groups, with the low-risk group being more suitable for receiving immunotherapy. Riskscore, age, grade, and radiotherapy were independent prognostic factors for HNSCC and were used to construct a nomogram, which had good clinical applicability.

**Conclusions:**

We successfully identified molecular subtypes and prognostic signature of HNSCC that are derived from GARGs, which can be used for the assessment of HNSCC prognosis and treatment responses.

**Supplementary Information:**

The online version contains supplementary material available at 10.1186/s12920-024-01823-9.

## Introduction

Head and neck squamous cell carcinoma (HNSCC) is a malignancy that originates from the squamous epithelial cells of the head and neck. It is among the most prevalent cancers in the head and neck region, including the mouth, throat, and nasopharynx. According to statistics, there were 890,000 new cases and 450,000 deaths in 2018 [1]. The incidence of HNSCC is expected to increase by 30% by 2030 [2], mainly due to factors such as smoking, alcohol consumption, and human papillomavirus infection [3]. Currently, most newly diagnosed HNSCC patients present with locally advanced disease and regional lymph node metastasis, which often leads to a high risk of recurrence and metastatic disease development [4]. There are currently various treatment options, such as surgery, radiotherapy, and chemotherapy, and in recent years, multidisciplinary comprehensive treatment and targeted immunotherapy have also made progress [5, 6]. However, due to the lack of early detection and postoperative recurrence in HNSCC, the 5-year survival rate of patients is less than 50% [7, 8]. Therefore, it is imperative to discover stable and reliable molecular features to assess patient prognosis and propose more effective treatment methods.

The Golgi apparatus (GA) is an organelle responsible for the sorting, modification, production, and transport of proteins and lipids. It plays crucial roles in various cellular processes, including cell migration, apoptosis, inflammation, autophagy, and stress response [9]. Additionally, GA promotes many cellular processes in cancer development, such as innate immune response, angiogenesis, tumor migration, and invasion [10, 11]. GA-targeted nanocarrier systems can modify the morphology of the GA, and have high specificity, low dosage, and minimal side effects, achieving satisfactory results in tumor treatment [12]. It has been observed that GA-related genes (GARGs) frequently mutate in tumors, leading to tumor metastasis and poor prognosis [13]. While GARGs-based models have been applied in the context of lung and liver cancers [14, 15], elucidating certain molecular mechanisms and prognostic factors for these malignancies, HNSCC presents with unique pathophysiological characteristics and therapeutic challenges. The development and progression of HNSCC involve intricate cellular processes such as cell migration, inflammation, and stress responses, which are closely tied to the functions of the Golgi apparatus. Consequently, the application of GARGs-based models of HNSCC is both necessary and holds promise for revealing disease-specific molecular mechanisms and prognostic factors pertaining to this particular cancer type.

**Fig. 1:**
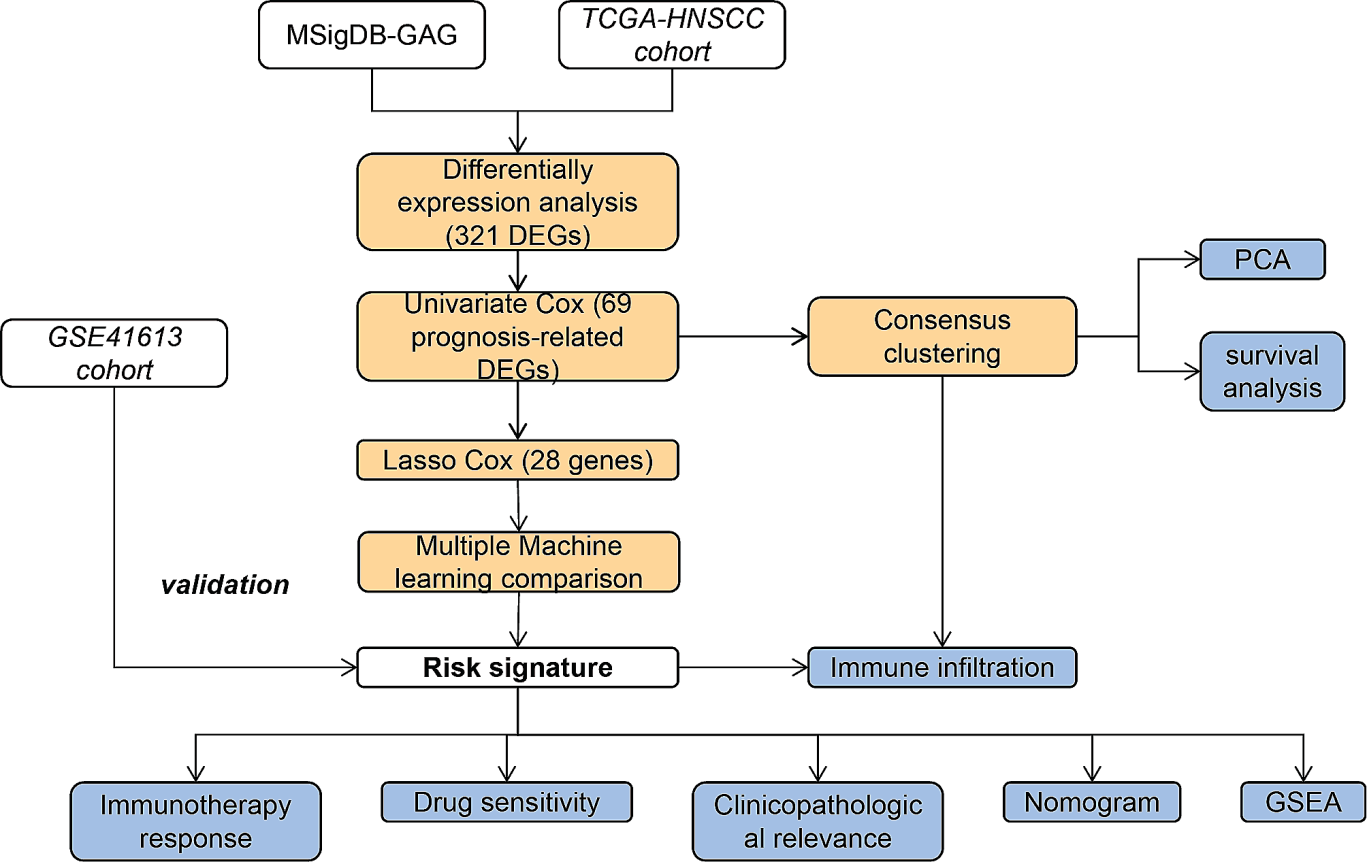
Flowchart of the research design in this study

In this study, we performed a comprehensive bioinformatics analysis of GARGs in HNSCC utilizing The Cancer Genome Atlas Program (TCGA, https://portal.gdc.cancer.gov/) and Gene Expression Omnibus (GEO, https://www.ncbi.nlm.nih.gov/gds/) databases. We identified HNSCC molecular subtypes based on GARGs and developed a GARG-based HNSCC prognosis risk model to offer clinical guidance for the treatment of HNSCC. The workflow diagram was illustrated in Fig. 1.

## Materials and methods

### Data collection and preprocessing

The transcriptome and clinical information of the TCGA-HNSCC cohort were obtained from the TCGA database. Cases with incomplete prognosis and clinical information were excluded from the cohort, and the remaining cases were used as the training set for model construction and molecular subtyping. The GSE41613 dataset from the GEO database, which includes transcriptome and clinical information of 96 HNSCC patients, was downloaded as the validation cohort. The GARGs were obtained from the GOCC_GOLGI_APPARATUS gene set available in the MSigDB (https://www.gsea-msigdb.org/gsea/msigdb/index.jsp) database.

### Consensus clustering of HNSCC

The limma package was used to conduct differential expression analysis of GARGs, with an adjusted *p*-value < 0.05 and|log (fold change)| < 1 as the threshold for selecting differentially expressed GARGs (deGARGs). Univariate Cox regression analysis was then used to identify deGARGs that were associated with HNSCC prognosis. Consensus clustering of the prognostic deGARGs was performed using the ConsensusClusterPlus R package to identify HNSCC molecular subtypes. Pam arithmetic and “pearson” distance were utilized to complete 500 bootstraps with every bootstrap containing ≥ 80% of TCGA-HNSCC dataset specimens. Cluster number k was between 2 and 6. The maximum cumulative distribution function (CDF) index was selected as the optimal k-value. Survival analysis was conducted to reveal prognostic differences between different molecular subtypes, and principal component analysis (PCA) was employed to verify this classification based on gene expression patterns among different subgroups.

### Construction and evaluation of GARGs-derived risk signature

The Lasso Cox regression analysis was employed to reduce the number of prognostic genes and construct a prognostic risk model using the glmnet package. The genes included in the model and the optimal value of the penalty coefficient λ were determined through running a 20,000-time 10-fold cross-validation probability using deviance for cross-validation. We employed a suite of 10 distinct machine learning algorithms, comprising random survival forest (RSF), elastic network(Enet), Lasso, Ridge, stepwise Cox, CoxBoost, partial least squares regression forCox (plsRcox), supervised principal components (SuperPC), generalised boostedregression modelling (GBM), and survival support vector machine (survival-SVM) [16]. These methods were utilized to construct predictive models on the TCGA-HNSCC cohort, which were subsequently validated on the independent GSE41613 dataset. Model performance was assessed using Harrell’s Consistency Index (C-index). The model that exhibited the highest average C-index across all datasets was designated as the optimal model. The training and validation sets were divided into high-risk and low-risk groups by the median risk score. Survival analysis was conducted to reveal prognostic differences between the two groups. Receiver operating characteristic curve analysis was used to evaluate the performance of the risk features in predicting 1, 3, and 5-year overall survival in HNSCC.

### Gene set enrichment analysis

The gene set enrichment analysis (GSEA) was performed using the clusterProfiler R package to identify Gene Ontology (GO) or Kyoto Encyclopedia of Genes and Genomes (KEGG) pathways [17] that were significantly suppressed or activated between high-risk and low-risk groups of patients. The significance was determined using an adjusted *p*-value < 0.05, and the results were visualized using a bubble plot.

### Immune infiltration analysis

The CIBERSORT R package was used to perform tumor tissue immune cell infiltration analysis on the TCGA-HNSCC cohort. This method calculates the infiltration of 22 immune cell types in HNSCC tumor tissue. Subsequently, inter-group differences were compared based on risk features and molecular subtypes.

### Treatment response analysis

The analysis of treatment response encompasses both immune therapy response and chemotherapy sensitivity analysis. The evaluation of immune therapy response is conducted through the utilization of the Tumor Immune Dysfunction and Exclusion (TIDE) algorithm, which computes TIDE score, cancer-associated fibroblasts (CAF) score, merck18 score, dysfunction score, and exclusion score to estimate the response to immune therapy. Standardized transcriptome data is uploaded to the TIDE website for computation. Chemotherapy sensitivity analysis is carried out using the pRRophetic R package, which selects the six most commonly used chemotherapy drugs in the TCGA-HNSCC cohort for sensitivity analysis and compares them between molecular subtypes and high-risk and low-risk groups.

### Construction and evaluation of nomogram

The independent prognostic factors of HNSCC were identified using multivariate Cox regression analysis, and a nomogram model was constructed using the identified independent prognostic factors. The model construction was performed using the rms R package, and model evaluation was conducted using calibration and decision curve analysis. The decision curve analysis was carried out using the rmda R package.

### Statistical analysis

R software (version 4.2.2) was utilized for data process, analysis and visualization. The Wilcoxon test was employed for intergroup difference analysis, while Kaplan-Meier survival curves and log-rank tests were utilized for survival analysis. A *p*-value < 0.05 was considered to be statistically significant.

## Results

### Molecular subtypes of HNSCC based on GARGs

**Fig. 2:**
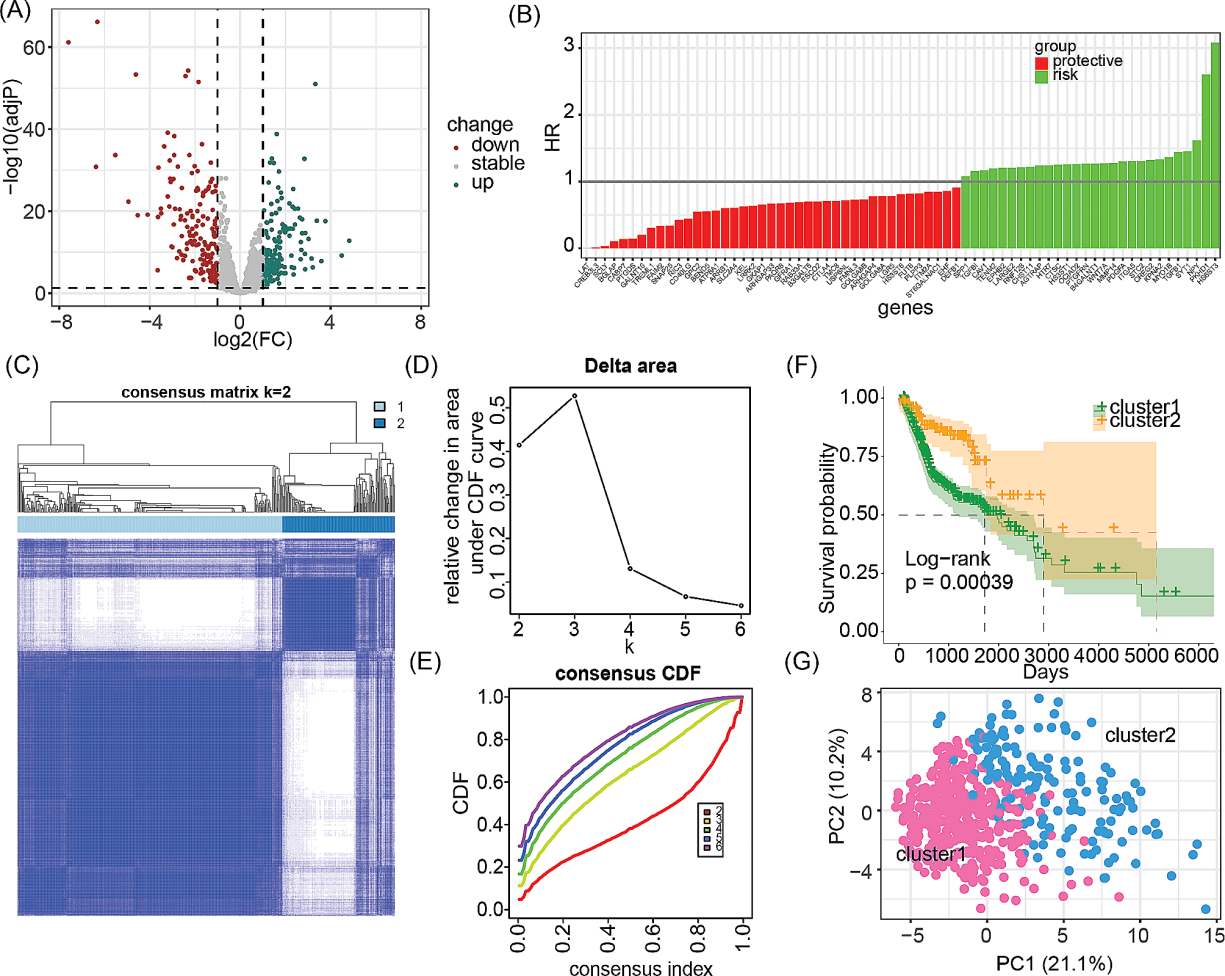
Molecular subtyping and prognostic analysis of GARGs in the TCGA-HNSCC cohort. (**A**) The volcano plot of the differentially expressed GARGs (deGARGs) in the TCGA-HNSCC cohort. (**B**) Distribution of hazard ratios of prognostic-related deGARGs. (**C**-**E**) Consensus clustering of the 69 prognostic-related deGARGs in the TCGA-HNSCC cohort. (**F**) Kaplan-Meier survival curve of molecular subtypes derived from GARGs. (**G**) Principal component analysis of the TCGA-HNSCC cohort based on prognostic-related deGARGs

A total of 1643 GARGs were obtained from the MSigDB database (Supplementary materials: Table S1). Differential analysis revealed that 321 of these genes were differentially expressed in HNSCC, with 162 upregulated and 159 downregulated (Fig. 2A, Supplementary materials: Table S2). Among these deGARGs, 69 were associated with HNSCC prognosis, of which 28 were risk genes and 41 were protective genes, as shown in Fig. 2B and Supplementary materials: Table S3. Using these prognostic deGARGs, two molecular subtypes (cluster 1 and cluster 2) of HNSCC were identified (Fig. 2C-E). Survival analysis demonstrated a significant difference in prognosis between the two clusters, with cluster 2 having a significantly better prognosis than cluster 1 (Fig. 2F). Principal component analysis revealed a clear boundary between cluster 1 and cluster 2 (Fig. 2G).

### GARGs-derived risk signature predicts HNSCC prognosis

**Fig. 3:**
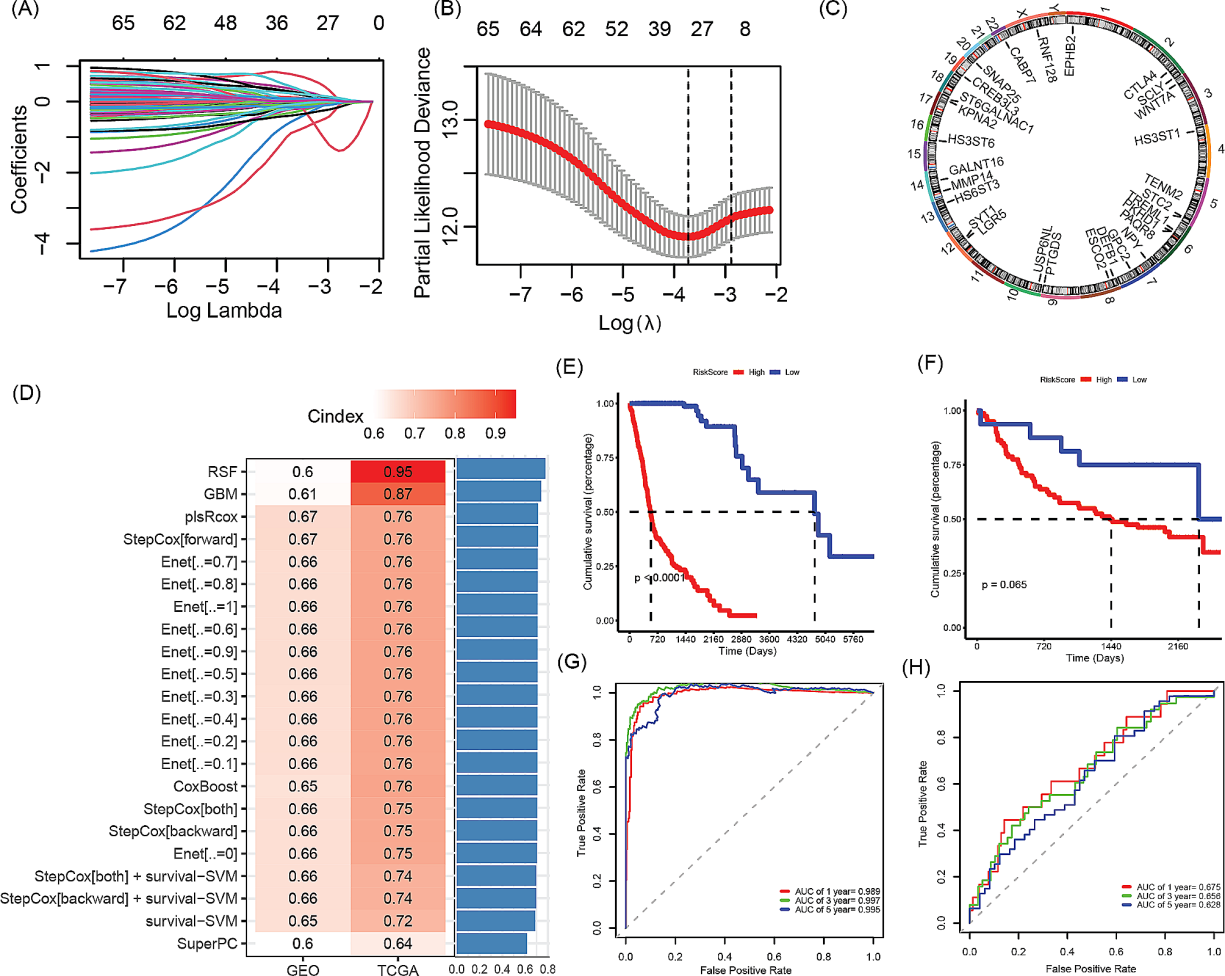
Construction and evaluation of prognostic risk features based on GARGs in the TCGA-HNSCC cohort. (**A**-**B**) Lasso Cox regression-based construction of prognostic signature derived from GARGs. (**C**) Chromosomal localization of 28 GARGs included in the risk signature. (**D**) C-index values based on machine learning prediction of the 28 GARGs in both TCGA-HNSCC and GSE41613 datasets. (**E**) Kaplan-Meier curves for high- and low-risk groups in the TCGA-HNSCC cohort. (**F**) Kaplan-Meier curves for high- and low-risk groups in the GSE41613 cohort. (**G**) ROC curves for the risk signature in the TCGA-HNSCC dataset. (**H**) ROC curves for the risk signature in the GSE41613 dataset

Lasso Cox regression analysis resulted in a set of 28 genes (Fig. 3A and **B**) whose chromosomal location was illustrated in Fig. 3C. Subsequently, a risk signature based on the 28 GARGs was accurately defined. In the TCGA-HNSCC cohort, we calculated the c-index for 10 individual predictive models and their combinations across all validation datasets (Fig. 3D). The superior model emerged as RSF, which displayed the highest average c-index at 0.775. Our analysis on HNSCC patients within both the TCGA-HNSCC and GSE41613 datasets indicated that higher risk scores were correlated with shortened survival times, as illustrated in Fig. 3E and F. In the TCGA-HNSCC dataset, the corresponding AUC values were recorded as 0.989, 0.997, and 0.996 (Fig. 3G), whereas for the GSE41613 dataset, the AUC values were 0.675, 0.656, and 0.628 (Fig. 3H).

### Association between GARGs-derived risk features and clinical pathological features

**Fig. 4:**
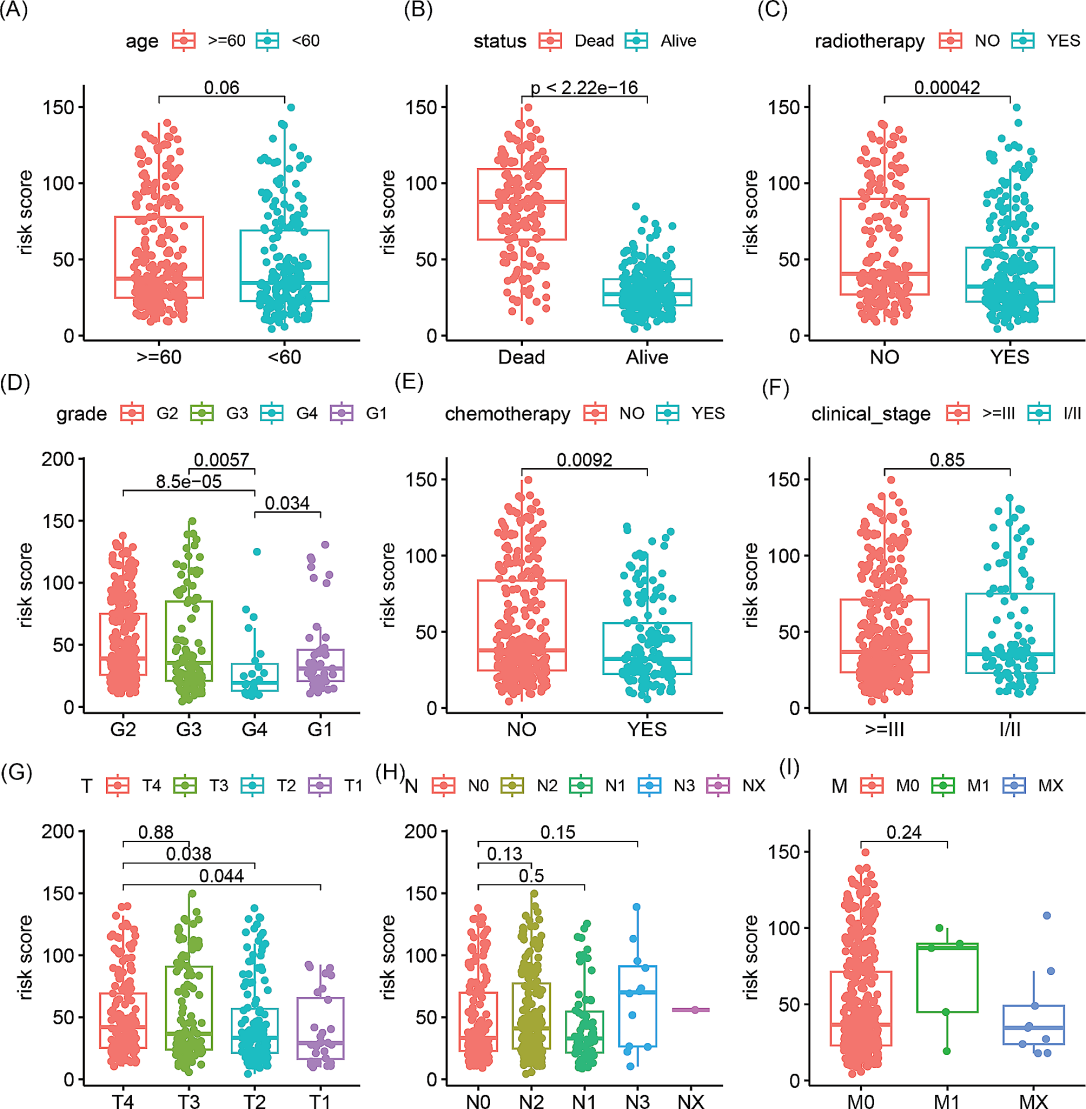
Differences in risk scores between different clinical and pathological feature groups. (**A**) Age. (**B**) Survival outcome. (**C**) Radiotherapy. (**D**) Grade. (**E**) Chemotherapy. (**F**) Clinical stage. (**G**) T stage. (**H**) N stage. (**I**) M stage

To explore the correlation between risk signature and clinical pathological features, comparisons of risk scores across various clinical pathological subgroups were performed. As shown in Fig. 4A-I, the risk scores of dead patients were significantly higher than those of surviving patients (*p* < 2.2e-16). Patients who did not receive radiation therapy had significantly higher risk scores compared to those who underwent radiation therapy (*p* = 0.00042). Furthermore, the risk scores of G4 grade were significantly lower than those of G1 (*p* = 0.034), G2 grade (*p* = 8.5e-05) and G3 (*p* = 0.0057), while the risk scores of patients who received chemotherapy were significantly lower than those who did not receive chemotherapy (*p* = 0.0092). Additionally, the risk scores of patients with T4 stage were significantly higher than those of patients with T2 (*p* = 0.038) and T1 (*p* = 0.044) stage. However, we did not observe any significant differences in risk scores among different age groups, clinical stages, and N and M stages.

### Abnormally expressed gene set between high-risk and low-risk groups

**Fig. 5:**
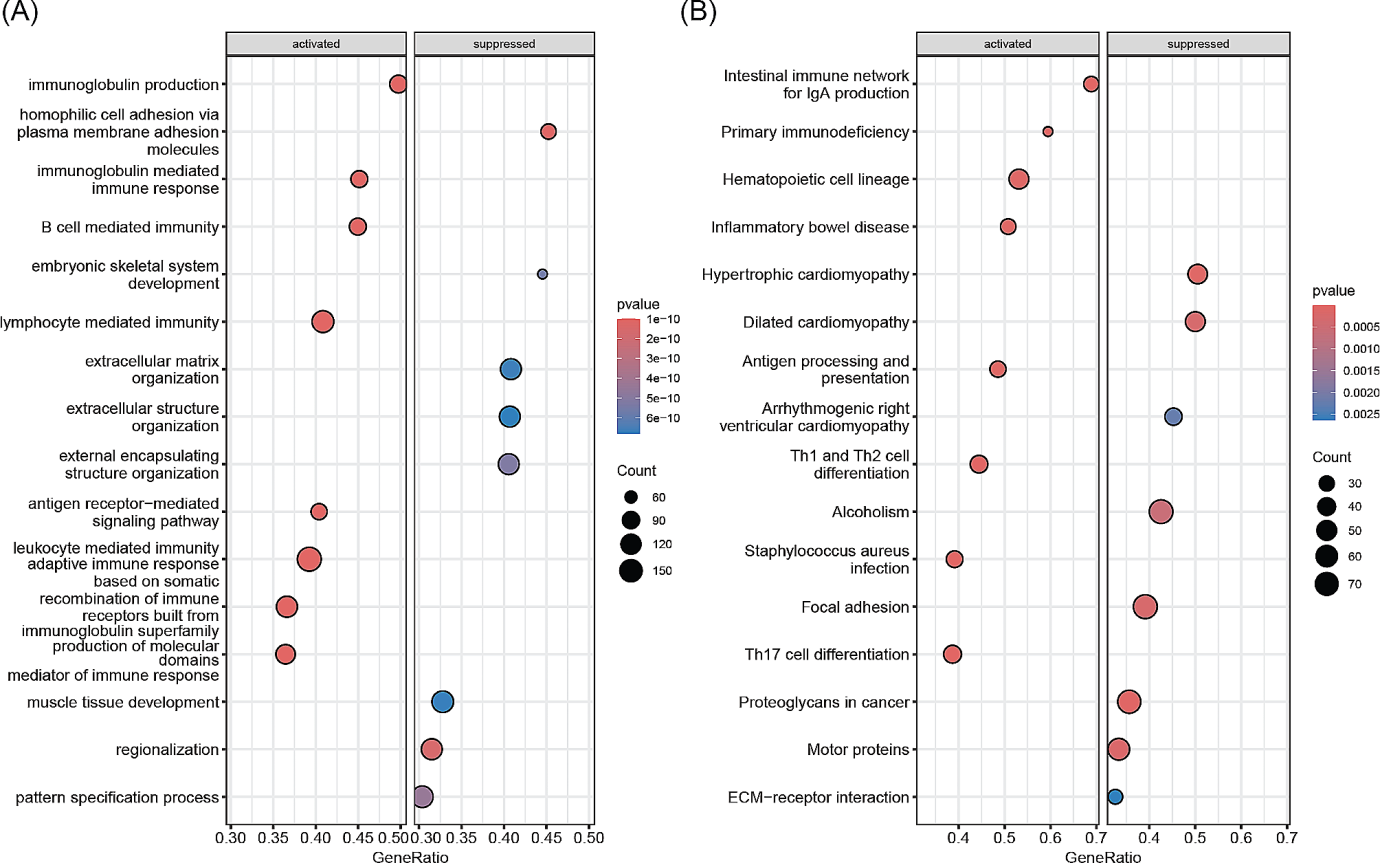
Gene set enrichment analysis between the high-risk and low-risk groups. (**A**) GO terms significantly activated and inhibited in the low-risk group compared to the high-risk group. (**B**) KEGG pathways significantly activated and inhibited in the low-risk group compared to the high-risk group

GSEA revealed that immune response-related biological processes were significantly activated in the high-risk group compared to the low-risk group. These processes included immune response, B cell mediated immunity, and immunoglobulin production, lymphocyte mediated immunity. Conversely, extracellular component-related processes were significantly suppressed in the high-risk group, such as organization of extracellular matrix, extracellular structure, and external encapsulating structure (Fig. 5A). Furthermore, T cell differentiation-related pathways were significantly activated in the high-risk group compared to the low-risk group, including Th1, Th2, and Th17 cells. However, pathways such as focal adhesion, proteoglycans in cancer, and ECM-receptor interaction were significantly suppressed (Fig. 5B).

### Differential immune infiltration between high-risk and low-risk groups

**Fig. 6:**
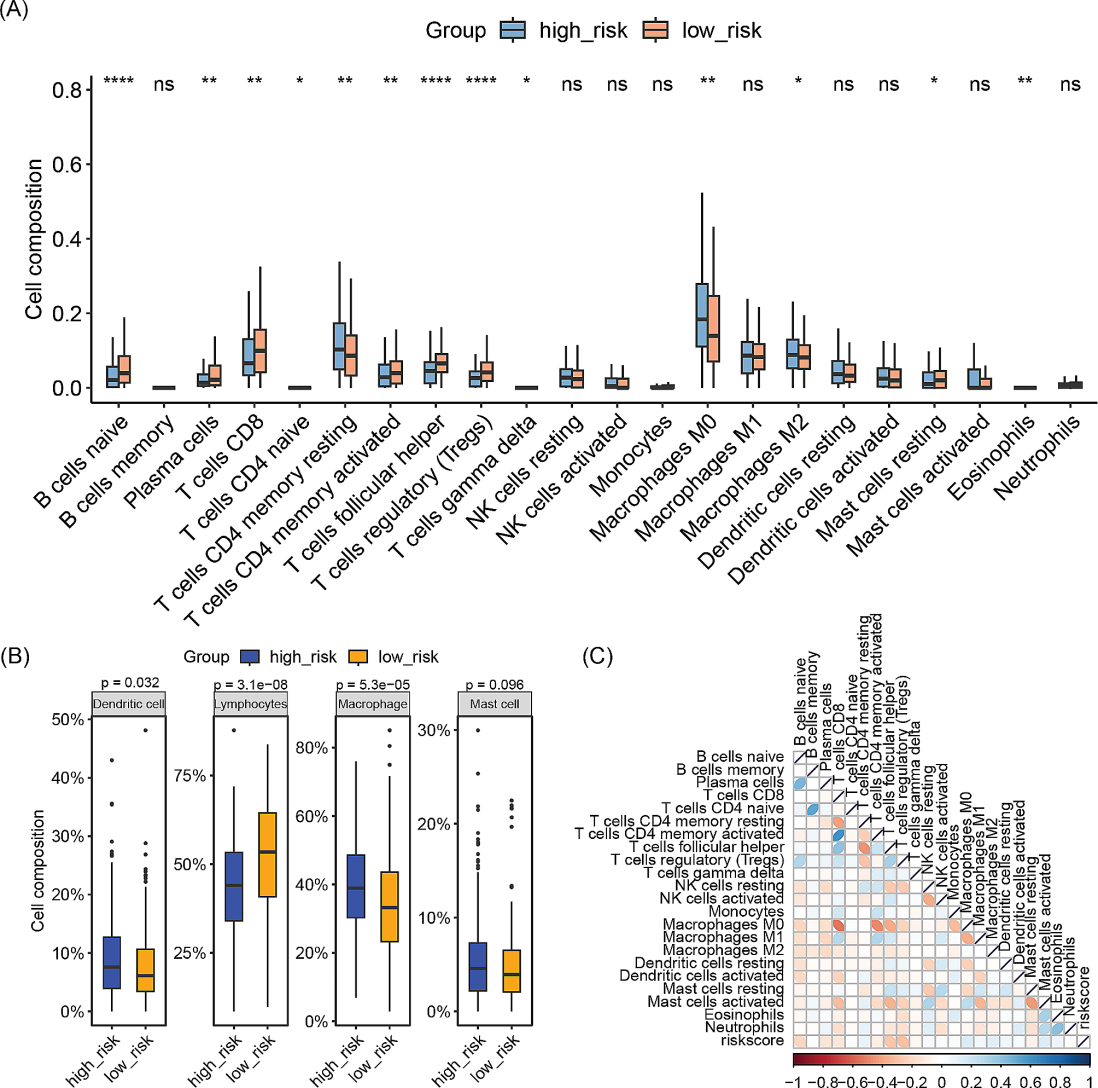
Comparison of immune infiltration differences between the high-risk and low-risk groups. (**A**) Comparison of immune cell infiltration in 22 immune cell types between the high-risk and low-risk groups. (**B**) Comparison of immune cell infiltration in 4 immune cell classifications between the high-risk and low-risk groups. (**C**) Correlation analysis between risk score and immune cell infiltration in 22 immune cell types. *p < 0.05, **p < 0.01, ***p < 0.001, ****p < 0.0001

In comparison to the high-risk group, the low-risk group exhibits significantly increased infiltration of B cells, CD8 T cells, activated CD4 T cells, follicular helper T cells (Tfh), regulatory T cells (Tregs), and resting mast cells in tumor tissues. Conversely, infiltration of resting CD4 T cells, M0 and M2 macrophages is significantly reduced in the low-risk group (Fig. 6A). Analysis of classified immune cells reveals that the low-risk group has higher levels of lymphocyte infiltration, while infiltration levels of macrophages and dendritic cells are lower in comparison to the high-risk group (Fig. 6B). Correlation analysis results between riskscore and 22 immune cells show a significant correlation between riskscore and most immune cell infiltrations (Fig. 6C).

### Differential immune therapy response and drug sensitivity between high-risk and low-risk groups

**Fig. 7:**
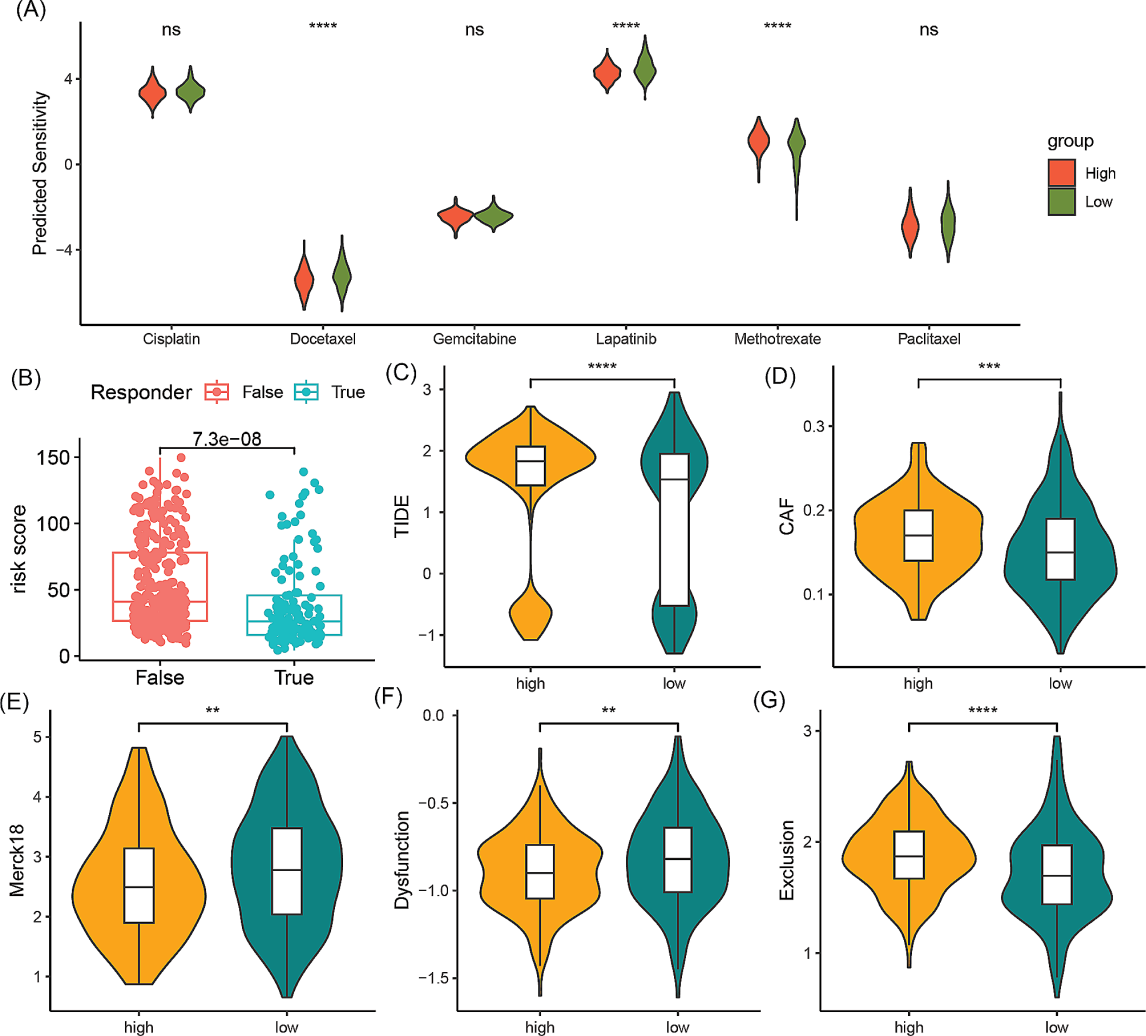
Evaluation of treatment response in the high-risk and low-risk groups. (**A**) Comparison of sensitivity to 6 chemotherapy drugs between the high-risk and low-risk groups. (**B**) Comparison of risk score between true responders and false responders in immunotherapy. (**C**-**G**) Comparison of TIDE, CAF, Merck18, Dysfunction, and Exclusion scores between the high-risk and low-risk groups. **p < 0.01, ***p < 0.001, ****p < 0.0001

The sensitivity of six drugs that are frequently used in the TCGA-HNSCC cohort were assessed. It was found that high-risk patients are more responsive to cisplatin, docetaxel, and lapatinib, while low-risk patients are more responsive to methotrexate (Fig. 7A). Accurately evaluating the response to immune therapy is critical for selecting appropriate patients and improving prognosis. Our analysis reveals that true responders to immune therapy have lower risk scores compared to false responders (Fig. 7B). Furthermore, compared to the high-risk group, low-risk patients exhibit lower TIDE, CAF, and exclusion scores, while having higher merck18 and dysfunction scores (Fig. 7C-G). These results suggest that low-risk patients have a better response to immune therapy and are more likely to benefit from it.

### Nomogram for HNSCC prognosis prediction

**Fig. 8:**
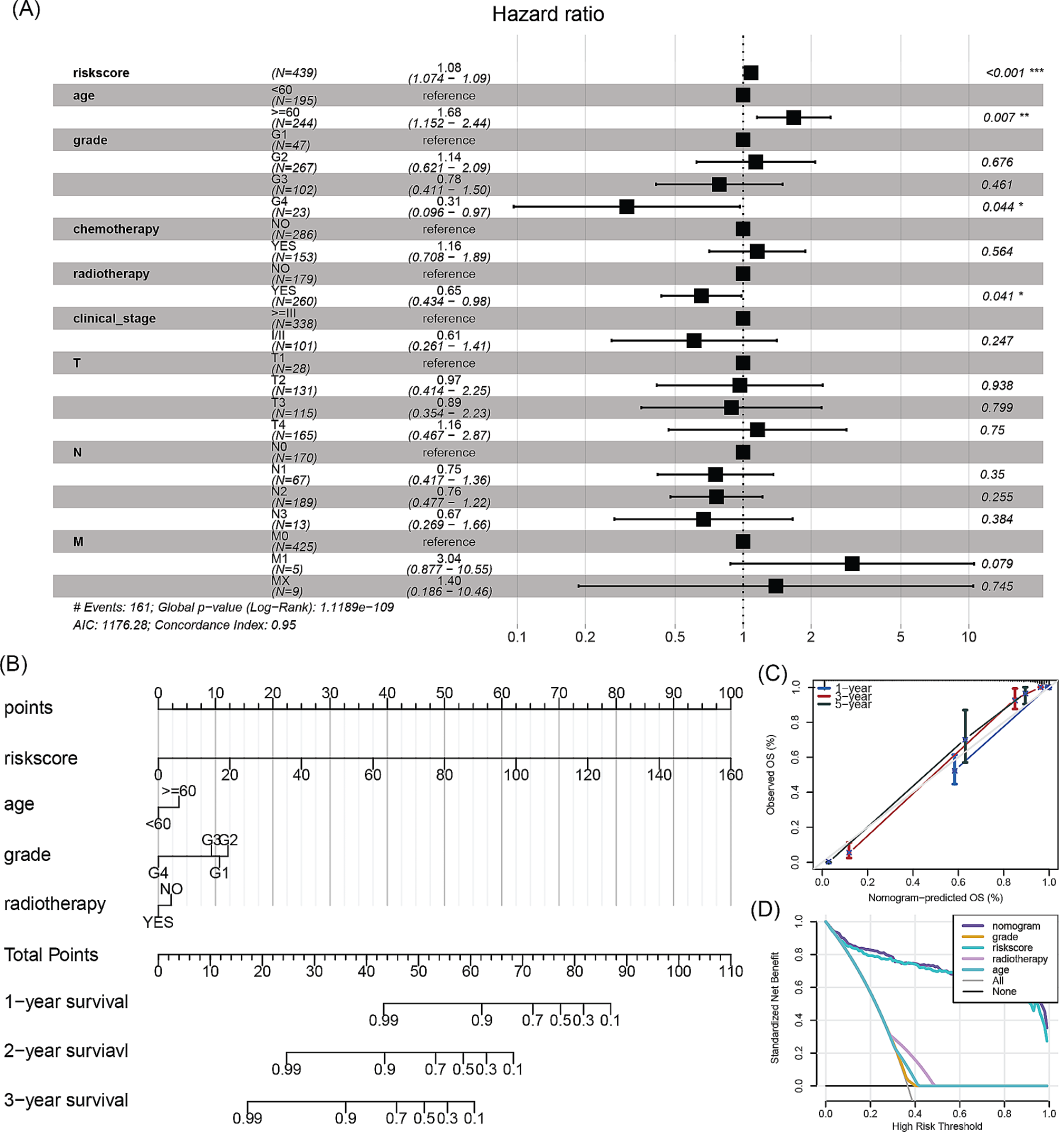
Construction and performance evaluation of a clinical nomogram model for HNSCC. (**A**) Multivariate Cox regression analysis of independent prognostic factors for HNSCC. (**B**) Nomogram composed of risk score, N stage, and radiotherapy for evaluating 1, 3, and 5-year overall survival in the TCGA-HNSCC cohort. (**C**) Calibration curve for evaluating the performance of the nomogram in predicting 1, 3, and 5-year overall survival. (**D**) Decision curve analysis for evaluating the performance of the nomogram and other prognostic factors in predicting 1-year overall survival. *p < 0.05, **p < 0.01, ***p < 0.001

To enhance the risk characterization derived from GARGs, we developed a nomogram to assess the overall survival of patients with HNSCC over 1, 3, and 5 years. Initially, a multivariate Cox regression analysis was conducted, which revealed that riskscore, age, grade, and radiotherapy were independent prognostic factors for patients with HNSCC (Fig. 8A). Subsequently, a nomogram was constructed using these three variables (Fig. 8B). Calibration curve analysis demonstrated that the nomogram had a good fit with the actual situation in predicting the 1, 3, and 5-year overall survival of patients with HNSCC (Fig. 8C). Compared to other prognostic factors, the nomogram had a superior net benefit in predicting the 1-year overall survival of patients with HNSCC (Fig. 8D).

## Discussion

With the advancement of treatment technology, the prognosis of HNSCC has significantly improved. However, its inherent heterogeneity can affect treatment response and thus treatment outcomes. Therefore, it is necessary to determine the accurate prognosis of HNSCC patients in order to provide personalized treatment plans. Previous studies have shown that the Golgi apparatus is involved in the occurrence and development of various tumors, including breast cancer [18], lung cancer [19], and hepatocellular carcinoma [20]. However, there is currently no systematic study on the prognostic value of GARGs in HNSCC and their relationship with drug sensitivity and immune therapy response.

In this study, we discovered that 321 GARGs exhibited differential expression in HNSCC, with 69 of them being significantly linked to HNSCC prognosis. By categorizing these genes based on their molecular subtypes, we identified two HNSCC molecular subtypes that displayed notable differences in both prognosis and immune infiltration. Moreover, through univariate and lasso Cox regression analyses, 28 GARGs were selected for the construction of a prognostic risk signature for HNSCC. For multi-factorial prognostic models, non-linear algorithms offer a promising approach to address the nonlinear interactions among risk factors [21–24]. Consequently, we assessed various machine learning algorithms and their combinations in constructing a risk model based on these 28 GARGs, and results demonstrated that the RSF model exhibited the most optimal predictive performance. Interestingly, the RSF model derived from GARGs is associated with drug sensitivity and immune therapy response, suggesting its potential utility in formulating personalized treatment plans.

Multiple GARGs encompassed in this prognostic signature exhibit significant associations with Golgi apparatus structure and function, and play integral roles in diverse facets of carcinogenesis. For example, cyclic AMP responsive element-binding protein 3-like 3 (CREB3L3), a member of the CREB3 transcription factor family, is intricately involved in endoplasmic reticulum and Golgi stress responses, thereby functioning as a pivotal regulator of cellular secretion capability and cargo specificity [25]. Extensive investigations have underscored its prognostic significance across various malignancies, including endometrial cancer [26] and gastric cancer [27]. Additionally, synaptosome-associated protein of 25 kDa (SNAP25), primarily localized to the plasma membrane, orchestrates regulated exocytosis of secretory vesicles [28]. Its regulatory influence on synaptic plasticity and glioma progression inhibition through GLS-mediated glutamine hydrolysis has been well-documented [29]. Moreover, the activation of Golgi Arf-like protein 1 (ARL1) is facilitated by synaptotagmin 1 (SYT1) [30], with ARL1 independently serving as a prognostic determinant for cutaneous melanoma, as higher ARL1 levels correspond to improved prognosis [31].

The immune infiltration of tumor tissue can have a significant impact on tumor development and treatment. Therefore, studying immune infiltration is crucial for tumor diagnosis, treatment, and prognosis evaluation. Herein, significant differences in immune cell infiltration were observed based on the HNSCC molecular subtypes of GARGs, which helps explain the significant differences in prognosis. In molecular subtypes with better prognosis, there was significant enrichment of B cells, CD8 cells, Tfh cells, and Treg cells. Studies have shown that germinal center tumor B cell infiltration in HNSCC patients indicates a good prognosis [32]. Enhancing the immune response of CD8 T cells is considered an important strategy in tumor immunotherapy. CD8 T cell infiltration changes with HNSCC local recurrence, and an increase in CD8 + T cell infiltration after recurrence indicates a good prognosis for HNSCC [33]. High infiltration of FoxP3^+^Treg is associated with better prognosis and can be used in combination with tumor staging and histological grading for prognosis evaluation of HNSCC patients [34]. These immune cell infiltration characteristics may be involved in the formation of drug sensitivity and immune therapy response in HNSCC, leading to differences between molecular subtypes and risk groups.

Despite the significant findings uncovered by this study, there remain several limitations. Firstly, the utilization of publicly available data from TCGA and GEO databases entails potential constraints. (i) the presence of sample selection bias may exert influence on the outcomes of biomarker screening endeavors. (ii) the challenge of multiple comparisons arises, giving rise to an augmented risk of false positive results attributable to chance discoveries. (iii) the dearth of external validation of the findings necessitates further independent validation and meticulous clinical studies to ascertain the efficacy and predictive value of the identified biomarkers. Secondly, the protein expression and biological functions of the genes comprising the model in HNSCC were inferred from literature and require further in vitro and in vivo studies for exploration. Finally, the clinical pathological features analyzed were not comprehensive enough, and additional features should be considered for future analysis and model optimization.

## Conclusion

This study innovatively employed machine learning to construct a GARG-based risk signature, uncovering the clinical relevance of Golgi apparatus-related genes in head and neck squamous cell carcinoma (HNSCC) prognosis. Analyzing public datasets, we identified differentially expressed GARGs linked to patient outcomes, enabling molecular subtyping into two prognostically distinct groups. The predictive power of this 28-GARG signature was validated, revealing clinicopathological associations, immune response differences, and treatment sensitivities between high- and low-risk patients. Our nomogram integrating riskscore, age, grade, and radiotherapy offers practical assistance for predicting overall survival in HNSCC, providing more guidance for personalized treatment decisions to improve patient care.

### Electronic supplementary material

Below is the link to the electronic supplementary material.

Supplementary Material 1

Supplementary Material 2

## Data Availability

The datasets used and/or analysed during the current study are available from NCBI Gene Expression Omnibus (GEO: GSE41613) https://www.ncbi.nlm.nih.gov/geo/, the cancer genome database (TCGA: HNSCC) https://portal.gdc.cancer.gov, and GARGs were available from the MSigDB https://www.gsea-msigdb.org/gsea/msigdb/index.jsp.
